# Morpho-Volumetric Changes of the Pharyngeal Airway With Traumatic Maxillofacial Injuries: A Retrospective Radiographic Study

**DOI:** 10.7759/cureus.47081

**Published:** 2023-10-15

**Authors:** Muath S Alassaf, Hamza K Khan, Osama A Habib, Ayyob E Aboalkhair, Hasan A Albeshir, Mahmood M Samman

**Affiliations:** 1 Orthodontics and Dentofacial Orthopedics, Taibah University, Madinah, SAU; 2 Oral and Maxillofacial Surgery, King Fahad General Hospital, Jeddah, SAU; 3 Oral and Maxillofacial Surgery, King Faisal Specialist Hospital and Research Centre, Madinah, SAU

**Keywords:** facial bone, facial trauma, traumatic facial injuries, pharyngeal airway volume, pharyngeal airway, maxillofacial trauma

## Abstract

Background: When dealing with traumatized patients, it is crucial to prioritize securing their airway. However, intubating someone who has sustained significant facial injuries can pose difficulties, as the narrow and altered shape of their upper airway may impede their ability to open their mouth. In light of this, the purpose of this study is to evaluate the volumetric and morphological alterations to the upper airway resulting from facial trauma by utilizing computed tomography (CT) scans.

Method: This is a single-centered retrospective analytical study. This study included CT scans of patients with traumatic facial injuries admitted to King Fahad Hospital in Madinah, Saudi Arabia. Study variables included age, gender, body mass index (BMI), fractured bones, airway symmetry, and airway volume. Using the 3D Slicer software (Slicer Community, USA), a three-dimensional model of the pharyngeal airway was constructed from the CT scan to evaluate symmetry and volume. IBM SPSS Statistics for Windows, version 23 (released 2013; IBM Corp., Armonk, New York, United States) was used to analyze data.

Results: Among the screened scans, 136 cases with traumatic facial injuries were included in the study. Age ranged from four to 91 years, with a mean of 28.26 (±14.9). Mandibular and zygomatic fractures were the most common, with 71 (52.2%) and 69 (50.7%) cases, respectively. The pharyngeal airway was symmetric in 111 (81.6%) cases and not symmetric in the other 25 (18.4%) cases. A significant association was found between the side of the fracture and airway asymmetry in mandibular fractures (p-value = 0.03). The total airway volume in the displaced mandibular fractures showed a statistically significant decrease (p-value = 0.019). The fracture sites were not statistically linked to airway asymmetry except for parasymphyseal and symphyseal fractures, with a p-value of 0.038 and 0.041, respectively.

Conclusion: The study findings suggest that the pharyngeal airway is not usually compromised in most facial bone fractures; however, bilateral displaced mandibular fractures have the potential to diminish the pharyngeal airway volume, especially in fractures involving the symphysis and parasymphysis area.

## Introduction

Traumatic injuries cause 5.8 million deaths each year, which is 10% of all deaths around the world [1]. Such injuries affect various parts of the body, with a high association of morbidity and mortality found in brain and maxillofacial injuries, which affect the quality of life of injured individuals [2]. Multiple studies were conducted worldwide to assess the etiology and pattern of maxillofacial traumatic injuries; the leading cause was road traffic accidents, accounting for 88.7-90.35% of the cases, with the mandible more affected than the maxilla and other facial fractures [3-8].

One of the most critical aspects of the management of trauma patients is establishing a secured airway. Any issue involving airway control can lead to serious illnesses or deaths. Regarding the patient's airway, maxillofacial trauma (MFT) presents a complex issue [9,10]. Airway narrowing or obstruction could be due to the posteroinferior displacement of a fractured maxilla, bilateral fracture of the anterior mandible that may cause posterior displacement of the tongue, and soft tissue swelling and edema resulting from trauma to the head and neck [9,11-15].

Intubation for the emergency management of severe MFT is usually difficult due to narrowing and changes in the morphology of the upper airway with limited mouth opening [9,11,16,17]. The upper airway is a complex structure that plays several vital roles in the human body, including respiration, food intake, and vocalization. It is formed and bounded by a combination of bony, cartilaginous, and soft tissues.

Computed tomography (CT) or cone-beam computed tomography (CBCT) can be used to assess airway volume and morphology. The lateral cephalometric radiograph is a two-dimensional radiograph that can also be used to assess the pharyngeal airway; however, it is less accurate compared to CT and CBCT [18]. The volume and morphology of a normal upper airway were studied by Abramson et al. [19] in which they measured the mean and minimum cross-sections at different areas with airway volume calculation and lateral-to-anteroposterior ratios. The airway volume was also studied in relation to skeletal discrepancies before and after orthognathic surgery, in which advancement of the mandible in cases with skeletal class II discrepancy showed an increase in the upper airway volume [20]. The changes in the upper airway were also evaluated in relation to palatal expansion, obstructive sleep apnea, and tumors of the head and neck area [21,22]. However, the radiographic changes in the upper airway in association with MFT have not been studied yet.

The aim of this study is to evaluate the changes in the upper airway in association with MFT in terms of volumetric and morphological changes using CT scans. The study hypothesized that the upper airway volume and morphology would be altered based on the involved fractures bone and its displacement.

## Materials and methods

Study design and population

This was a single-center retrospective analytical study. This study included CT scans of patients with MFT admitted to King Fahad Hospital in Madinah, Saudi Arabia, from January 2018 to September 2023. Scans must show the skull and cervical vertebrae to the level of the fifth one. No restrictions for age, gender, or nationality were applied. Cases with pathologies or syndromes that might affect the pharyngeal airway space were excluded. In addition, CT scans with blurred images, poor quality, thick slices, or artifacts were excluded. Intubated cases and scans showing fractures in the cervical vertebrae were also excluded. Among the screened 864 scans, 136 scans were included according to the abovementioned criteria.

Demographic data and classification of fractures

Data were collected into a spreadsheet with three sections: the first section included the age, gender, and body mass index (BMI). Age was divided into three categories: below the age of 20 years, 20 to 40 years, and above 40 years. This section was filled according to the patient's medical record. The second section included the type of fractures in terms of the involved bone (mandible, maxilla, zygoma, frontal bone, and nasal bone), side (right, left, or bilateral), and displacement. For each fracture, the anatomic site or sites were described. The CT report written by the radiology consultant was used to fill the second section; all described fractures were confirmed on the CT scan by two investigators. The third section of the data collection sheet included the airway symmetry (symmetric or asymmetric) and airway volume.

Airway morphology and volume

**Figure 1:**
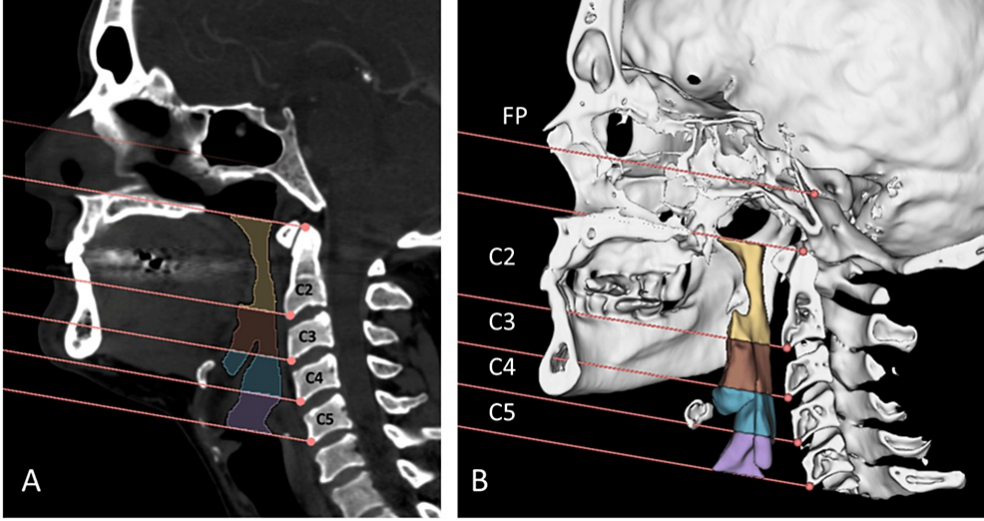
Construction of the 3D model of the pharyngeal airway. (A) sagittal view used to orient the lines dividing the parts of the pharyngeal airway parallel to the Frankfort plane (FP). (B) 3D model of the bones (white) with the segments of the pharyngeal airway (colored).

**Figure 2:**
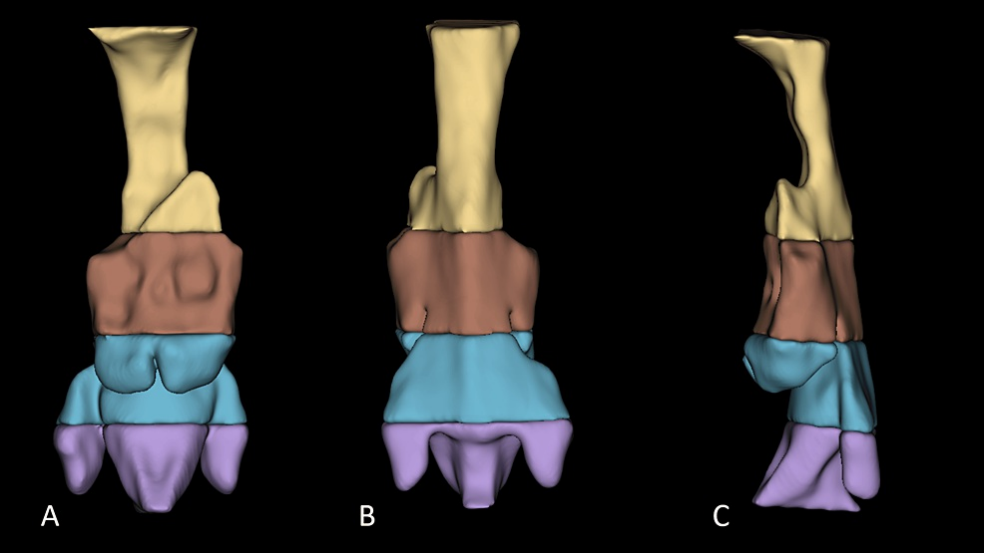
3D model of the pharyngeal airway showing asymmetry. (A) anterior view, (B) posterior view, (C) side view.

All CT scans were obtained by the Aquilion Prime SP CT scanner (Canon Medical Systems, Ohtawara, Japan) in a supine position. Using the 3D Slicer software (Slicer Community, USA), a three-dimensional (3D) model of the pharyngeal airway was constructed from the CT scan [23]. 3D Slicer is a free source software reported to have good threshold sensitivity [24]. The pharyngeal airway was defined as the space from the level of the hard palate to the level of the fifth cervical vertebra. As landmarks from facial bones might be altered due to the MFT, in this study, the pharyngeal airway is considered from the level of the superior border of the odontoid process of the axis vertebra to the inferior border of the fifth vertebra using a line parallel to the Frankfort plane (imaginary line from the external auditory meatus to the infraorbital rim). Guided by the sagittal view at midline, the airway space is divided into four parts according to the cervical vertebrae behind it. C2 represents the part anterior to the first and second vertebrae. C3, C4, and C5 each correspond to the space anterior to the vertebrae. These parts are segmented based on planes at the level of the anteroinferior border of the vertebrae parallel to the Frankfort plane, as shown in Figure 1. This 3D model was used to evaluate the morphology by assessing the symmetry of the airway from anterior and posterior views, as shown in Figure 2. The volume of the segments was calculated using segment statistics extension from the software in cubic centimeters (cm^3^). The total volume was calculated as the sum of the C2, C3, C4, and C5 segments.

Two highly skilled investigators, each with more than five years of experience in CT analysis and interpretation, shared software instructions and conducted necessary manipulations to guarantee precise data collection, calibration, and standardized interpretation. Prior to conducting the actual evaluation of the CT scans, each examiner evaluated 10 CT scans that were chosen from extra data that were excluded from the study results. This rigorous approach ensured a more scientific and reliable outcome.

Data analysis

The data was analyzed using the IBM SPSS Statistics for Windows, version 23 (released 2013; IBM Corp., Armonk, New York, United States). Sample characteristics were summarized through descriptive analysis. For variables that followed a normal distribution (Kolmogorov-Smirnov; p>0.05), the mean and standard deviations (M±SD) were presented. For non-normally distributed data, the median and interquartile range (IQR) were used. Qualitative variables were analyzed based on frequency and percentages. Parametric tests, such as Student or paired T-test, were used for normally distributed data, while non-parametric tests, such as the U-test, were used for non-normally distributed data. A chi-squared test was used to compare between groups for qualitative variables. A p-value of 0.05 or less was considered significant.

Ethical considerations

Each subject was assigned an anonymous code, and all data were recorded in an Excel sheet (Microsoft Excel, Microsoft, USA) with a secure password. In addition, the computer was password-protected. This study was approved by the Institutional Review Board (IRB), General Directorate of Health Affairs in Madinah, Saudi Arabia (approval no.: 23-076). As this study used already existing radiographic scans, the waiver of consent was requested and approved by the IRB.

## Results

Among the screened scans, 136 cases with MFT were included in the study. Age ranged from four to 91 years, with a mean of 28.26 (±14.9). The 20-to-40-year category was dominant, with 70 (51.5%) cases. The male gender accounted for 109 (80.1%). The BMI mean was 25.65 (±5.17), with 78 (57.4%) being healthy. Table 1 summarizes the sample characteristics in terms of age groups, gender, and BMI. No significant differences were noted between groups (p-value > 0.05).

**Table 1:** Distribution of age and body mass index (BMI) among the studied population (n = 136)

Variable	Male (%)	Female (%)	Total (%)	P-value
Age groups				0.67
Below 20	32 (23.5%)	7 (5.1%)	39 (28.7%)	
20-40	57 (41.9%)	13 (9.6%)	70 (51.5%)	
Above 40	20 (14.7%)	7 (5.1%)	27 (19.9%)	
BMI class				0.92
Underweight	10 (7.4%)	2 (1.5%)	12 (8.8%)	
Healthy	61 (44.9%)	17 (12.5%)	78 (57.4)	
Overweight	22 (16.2%)	5 (3.7%)	27 (19.9%)	
Obese	16 (11.8%)	3 (2.2%)	19 (14%)	

Mandibular and zygomatic fractures were the most common, with 71 (52.2%) and 69 (50.7%) cases, respectively. According to the involved side, bilateral fractures occurred in 31 (43.7%) cases of mandibular fractures, while 15 (24.2%) and seven (10.1%) cases only were bilateral fractures for the maxilla and zygoma. The right and left sides were equally involved in the mandibular fractures (20, 28.2%). In the zygomatic and maxillary bone fractures, the left side is more commonly affected, with 26 (41.9%) and 33 (47.8%) cases, respectively. Regarding displacement, mandibular fractures were more commonly displaced compared to other bones, with 47 (66.2%) cases of displacement in the mandibular fractures. Table 2 summarizes the distribution of the cases in terms of fractured bones, side, and displacement.

**Table 2:** Distribution of maxillofacial fractures according to the involved bone, side, and displacement (n = 136)

Variable	Bilateral (%)	Lt (%)	Rt (%)	Displaced (%)	Total (% of 136)
Mandible	31 (43.7)	20 (28.2)	20 (28.2)	47 (66.2)	71 (52.2)
Maxilla	15 (24.2)	26 (41.9)	21 (33.9)	18 (29.0)	62 (45.6)
Zygoma	7 (10.1)	33 (47.8)	29 (42)	33 (47.8)	69 (50.7)
Frontal bone	10 (50)	5 (25)	5 (25)	5 (25)	20 (14.7)
Nasal bone	25 (80.6)	1 (3.2)	6 (19.4)	6 (19.4)	31 (22.8)

**Figure 3:**
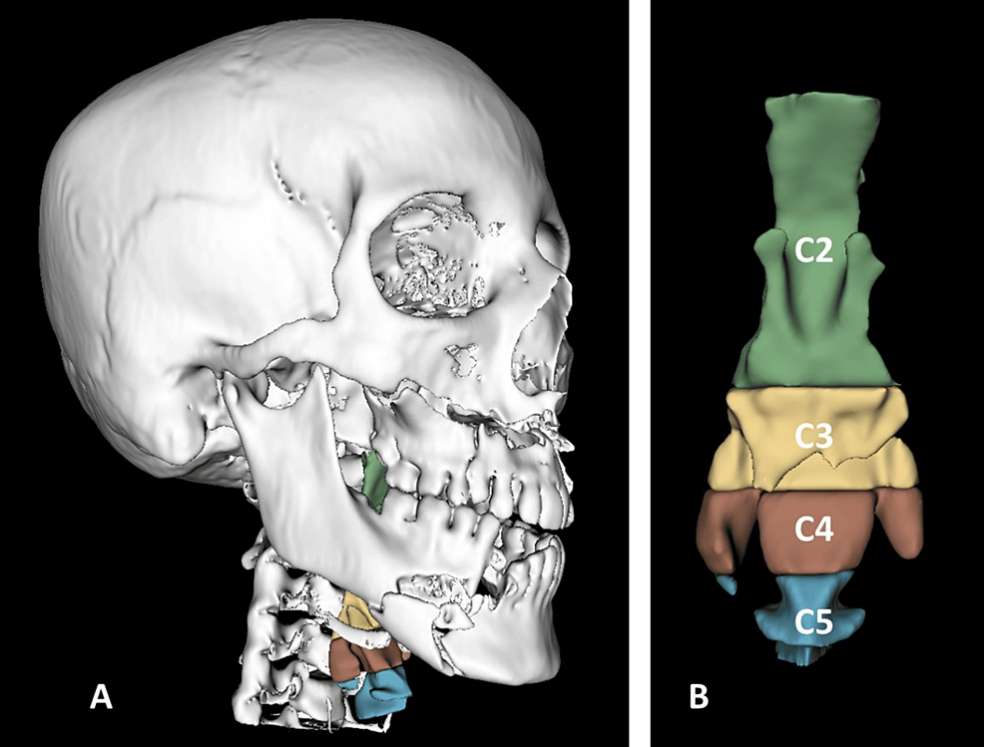
(A) 3D model of the facial bone with multiple facial fractures. (B) The constructed 3D model of the pharyngeal airway and segments marked asymmetry at the level of C3.

The pharyngeal airway was symmetric in 111 (81.6%) cases and not symmetric in the other 25 (18.4%) cases. The means among the studied cases based on the airway symmetry are presented in Table 3. Comparison between means was statistically significant at the level of C3, in which the asymmetric airway had a lower mean of 2.37 (±0.96) than the symmetric one (3.58, ±2.15). Figure 3 shows the 3D model of the pharyngeal airway and fractured facial bones with marked C3 and other segments.

**Table 3:** Mean and standard deviation (SD) for the upper airway volume in (cm3) based on the symmetry (n = 136) * P-value less than 0.05 after the U-test.

Level	Aymmetric		Symmetric		P-value
	Mean	SD	Mean	SD	
Total volume	12.46	5.30	15.09	6.98	.067
Vol. C2	4.84	2.35	5.64	3.06	.159
Vol. C3	2.37	0.96	3.58	2.15	.016*
Vol. C4	2.84	1.13	3.49	1.95	.241
Vol. C5	2.42	2.10	2.39	1.54	.452

The correlation between the airway volume and age revealed a positive association at all levels, as shown in Table 4. However, no correlation was found with the sample BMI. The comparison between the airway symmetry and type of fractured bone revealed a statistically significant difference (p-value = 0.008) for mandibular fractures as it accounted for 19 (26.8%) cases, compared to 12 (19.5%), 13 (18.8%), two (10%), and three (9.4%) cases for maxilla, zygoma, frontal, and nasal bone, respectively. A summary of the fractures and airway symmetry is shown in Table 5.

**Table 4:** Correlation between age, BMI, and airway volumes (n = 136) * P-value less than 0.05; ** P-value less than 0.01

Variables	Correlation test	Total volume	Vol. C2	Vol. C3	Vol. C4	Vol. C5
Age	Pearson's correlation	.301	.192	.277	.323	.252
	Sig. (2-tailed)	.000^**^	.025^*^	.001^**^	.000^**^	.006^**^
BMI	Pearson's correlation	-.029	-.073	.006	.018	.042
	Sig. (2-tailed)	.738	.401	.949	.832	.654

**Table 5:** Distribution of types of maxillofacial fractures based on the airway symmetry (n = 136) * P-value less than 0.05 after the chi-squared test

Variable	Aymmetric (%)	Symmetric (%)	Total (% of 136)	P-value
Mandible	19 (26.8)	52 (73.2)	71 (52.2)	0.008*
Maxilla	12 (19.4)	50 (80.6)	62 (45.6)	0.789
Zygoma	13 (18.8)	56 (81.2)	69 (50.7)	0.889
Frontal bone	2 (10)	18 (90)	20 (14.7)	0.295
Nasal bone	3 (9.4)	29 (90.6)	32 (23.5)	0.133

More details about the anatomic site, side, and displacement of mandibular fractures are shown in Table 6. It shows that there is a significant association between the side of the fracture and airway asymmetry in mandibular fractures (p-value = 0.03) with Cramer’s V value of 0.25 and approximate significance of 0.037 (df = 3). For displacement, no statistical differences were found in relation to the airway asymmetry. However, the comparison of the total airway volume between the displaced and non-displaced mandibular fractures showed a statistically significant difference (p-value = 0.019) after the U-test. The fracture sites were not statistically linked to the airway asymmetry except for parasymphyseal and symphyseal fractures, with a p-value of 0.038 and 0.041, respectively.

**Table 6:** Distribution of the side, displacement, and site of mandibular fractures based on the airway symmetry (n = 71) * Phi and Cramer’s V value is 0.250 with an approximate significance of 0.037 (df = 3). ** Phi and Cramer’s V value is 0.178 with an approximate significance of 0.038 (df = 1). *** Phi and Cramer’s V value is 0.175 with an approximate significance of 0.041 (df = 1).

Variable	Asymmetric (%)	Symmetric (%)	Total	P-value
Fracture side and displacement				
Bilateral	8 (25.8)	23 (74.2)	31 (43.7)	0.037*
Left	7 (35)	13 (65)	20 (28.2)	
Right	4 (20)	16 (80)	20 (28.2)	
Displacement	11 (23.4)	36 (76.6)	47 (66.2)	0.272
Fracture site				
Condyle	7 (25.9)	20 (74.1)	27 (38)	0.258
Coronoid	1 (33.3)	2 (66.7)	3 (4.2)	0.499
Angle	6 (30)	14 (70)	20 (28.2)	0.634
Body	7 (25.9)	20 (74.1)	27 (38)	0.109
Parasymphysis	7 (35)	13 (65)	20 (28.2)	0.038**
Symphysis	3 (42.9)	4 (57.1)	7 (9.9)	0.041***

## Discussion

MFTs are common, with road traffic accidents being the most common cause [2,5,6,15]. The consequences of MFTs vary and negatively affect the quality of life. Most cases require management under general anesthesia with oropharyngeal or nasopharyngeal intubation, which is a complicated issue in cases of severe MFTs [9,11,14,25]. In MFTs, the airway might get compromised or obstructed by means of foreign bodies, laryngeal injuries, hematoma formation, edema in fracture areas, and displacement of anatomic structures, such as the tongue in cases of bilateral parasymphyseal fractures [26]. Understanding the changes in the pharyngeal airway with different bone fractures in the facial region might ease or partly simplify this issue. This study hypothesizes that the morphology and volume of the pharyngeal airway could be altered due to maxillofacial injuries involving the bones around the airway. The results of this study suggest that the airway morphology and volume differ based on the fractured bone, its side, and the site within the bone.

Age, gender, and distribution of fractures

Most patients of the included cases were male, with age between 20 and 40 years. This is consistent with most worldwide studies investigating MFTs. This might be attributable to the increased activities in the young adults’ age [2,27]. The distribution of the cases shows that the mandible is the most commonly fractured bone (71, 52.2%), followed by the zygomatic bone (69, 50.7%). This goes in line with the findings of multiple studies [6-8,27]. For mandibular fractures, our study found equal involvement for condylar and body fractures, with 27 (38%) cases for each, followed by parasymphysis and body fractures, with 20 (28.2) cases for each. Condylar fractures, as the most common involved site, have been reported by Samman et al. and others [4,6,28]. In addition, fractures of the mandibular body were also reported as the most common fractured mandible by Rabi and Khateery [7]. For mid-face fracture, the zygoma was the most commonly fractured bone, followed by the maxilla; these findings are consistent with the literature [6,8].

Airway volume compared to previous studies

The airway is a vital passage for the aerodigestive tract. Airway maintenance is a crucial step in all emergency protocols [10]. The airway volume increases with age, as stated by Chuang et al. and Abramson et al. [19,29], who evaluated the airway changes with growth using CT scans. In this study, a positive correlation was also found between age and all levels of airway volume (Table 4). Regarding the BMI and airway volume, a study by Abramson et al. [19] showed an inverse relation between BMI and airway linear measurements, in which as the BMI increases, the airway linear measurements decrease. Our data presented similar findings but without a statistical difference (Table 4) [19].

Airway asymmetry and trauma types

This study aimed to evaluate the morphological and volumetric changes of the pharyngeal airway in cases of MFT. Since the included cases were not intubated and normally underwent CT imaging, their airway was not obstructed; however, it could be compromised, and this study evaluated the extent of changes in the airway volume and morphology.

The morphologic changes were assessed by the asymmetry of the 3D airway model. Of the 136 cases, the airway was not symmetric in 25 cases. The distribution of these cases is shown in Table 5, and the asymmetry cases were mostly in mandibular fractures with statistical significance (p-value = 0.008). The in-depth evaluation of mandibular fractures and airway asymmetry showed that bilateral mandibular fractures showed asymmetry more than unilateral cases with statistical significance (p-value = 0.037). As per the site, fractures in the symphysis and parasymphysis region also showed statistical differences for airway asymmetry compared to the other sites, with a p-value of 0.041 and 0.038, respectively. Fractures of the mandibular symphysis and parasymphysis region were reported to cause airway obstruction in multiple case reports. Most suggest the involvement of genial tubercles in the fractured segment [30]. Cases with airway obstruction are likely to be intubated at the time of CT imaging and, hence, not included in this study; nevertheless, the decrease in volume was observed in symphyseal and parasymphyseal fractures.

In terms of volume, a comparison between symmetric and asymmetric airway volumes showed that there is a significant difference at the level of C3, in which the C3 volume is smaller in cases of airway asymmetry (p-value = 0.016). Comparing the airway volumes showed that in displaced mandibular fractures, the total volume decreased (p-value = 0.019).

Limitations and recommendations

This study has certain limitations. The relatively small sample size and the single-center nature of the study may limit the generalizability of the findings. However, the establishment of a standardized method for measuring the pharyngeal airway in MFT patients lays the foundation for future research in this area. It is important to note that the absence of a control group from non-trauma cases reduces the comparability of the results. Nevertheless, cases with symmetric airways were utilized as an internal control group. In addition, the exclusion of intubated cases, which could provide insights into the risk of airway obstruction in MFT, is another limitation. Future studies are recommended to overcome these limitations by incorporating larger sample sizes from multiple centers to yield more generalizable and robust findings.

## Conclusions

The study findings suggest that the pharyngeal airway is not usually compromised in most facial bone fractures; however, bilateral displaced mandibular fractures have the potential to diminish the pharyngeal airway volume, especially in fractures involving the symphysis and parasymphysis area.
